# Genome-Wide Meta-Analysis of Homocysteine and Methionine Metabolism Identifies Five One Carbon Metabolism Loci and a Novel Association of *ALDH1L1* with Ischemic Stroke

**DOI:** 10.1371/journal.pgen.1004214

**Published:** 2014-03-20

**Authors:** Stephen R. Williams, Qiong Yang, Fang Chen, Xuan Liu, Keith L. Keene, Paul Jacques, Wei-Min Chen, Galit Weinstein, Fang-Chi Hsu, Alexa Beiser, Liewei Wang, Ebony Bookman, Kimberly F. Doheny, Philip A. Wolf, Michelle Zilka, Jacob Selhub, Sarah Nelson, Stephanie M. Gogarten, Bradford B. Worrall, Sudha Seshadri, Michèle M. Sale, the Genomics and Randomized Trials Network, the Framingham Heart Study

**Affiliations:** 1 Center for Public Health Genomics, University of Virginia, Charlottesville, Virginia, United States of America; 2 Cardiovascular Research Center, University of Virginia, Charlottesville, Virginia, United States of America; 3 Department of Biostatistics, Boston University School of Public Health, Boston, Massachusetts, United States of America; 4 The Framingham Heart Study, Framingham, Massachusetts, United States of America; 5 Department of Public Health Sciences, University of Virginia, Charlottesville, Virginia, United States of America; 6 Department of Biology, East Carolina University, Greenville, North Carolina, United States of America; 7 Center for Health Disparities Research, East Carolina University, Greenville, North Carolina, United States of America; 8 Jean Mayer USDA Human Nutrition Research Center on Aging and Friedman School of Nutrition Science and Policy, Tufts University, Boston, Massachusetts, United States of America; 9 Department of Neurology, Boston University School of Medicine, Boston, Massachusetts, United States of America; 10 Department of Biostatistical Sciences, Wake Forest School of Medicine, Winston-Salem, North Carolina, United States of America; 11 Department of Molecular Pharmacology and Experimental Therapeutics, Mayo Clinic College of Medicine, Rochester, Minnesota, United States of America; 12 National Human Genome Research Institute, Bethesda, Maryland, United States of America; 13 Center for Inherited Disease Research, Johns Hopkins University, Baltimore, Maryland, United States of America; 14 Department of Biostatistics, University of Washington, Seattle, Washington, United States of America; 15 Department of Neurology University of Virginia, Charlottesville, Virginia, United States of America; 16 Department of Medicine, University of Virginia, Charlottesville, Virginia, United States of America; 17 Department of Biochemistry and Molecular Genetics, University of Virginia, Charlottesville, Virginia, United States of America; University of Texas MD Anderson Cancer Center, United States of America

## Abstract

Circulating homocysteine levels (tHcy), a product of the folate one carbon metabolism pathway (FOCM) through the demethylation of methionine, are heritable and are associated with an increased risk of common diseases such as stroke, cardiovascular disease (CVD), cancer and dementia. The FOCM is the sole source of *de novo* methyl group synthesis, impacting many biological and epigenetic pathways. However, the genetic determinants of elevated tHcy (hyperhomocysteinemia), dysregulation of methionine metabolism and the underlying biological processes remain unclear. We conducted independent genome-wide association studies and a meta-analysis of methionine metabolism, characterized by post-methionine load test tHcy, in 2,710 participants from the Framingham Heart Study (FHS) and 2,100 participants from the Vitamin Intervention for Stroke Prevention (VISP) clinical trial, and then examined the association of the identified loci with incident stroke in FHS. Five genes in the FOCM pathway (*GNMT* [p = 1.60×10^−63^], *CBS* [p = 3.15×10^−26^], *CPS1* [p = 9.10×10^−13^], *ALDH1L1* [p = 7.3×10^−13^] *and PSPH* [p = 1.17×10^−16^]) were strongly associated with the difference between pre- and post-methionine load test tHcy levels (ΔPOST). Of these, one variant in the *ALDH1L1* locus, rs2364368, was associated with incident ischemic stroke. Promoter analyses reveal genetic and epigenetic differences that may explain a direct effect on *GNMT* transcription and a downstream affect on methionine metabolism. Additionally, a genetic-score consisting of the five significant loci explains 13% of the variance of ΔPOST in FHS and 6% of the variance in VISP. Association between variants in FOCM genes with ΔPOST suggest novel mechanisms that lead to differences in methionine metabolism, and possibly the epigenome, impacting disease risk. These data emphasize the importance of a concerted effort to understand regulators of one carbon metabolism as potential therapeutic targets.

## Author Summary

Elevated homocysteine (tHcy) is strongly associated with risk for common disorders such as stroke, cardiovascular disease and Alzheimer disease. Lowering tHcy levels has proven to have variable success in reducing clinical risk, so the question remains, “Are we correctly targeting these disorders by lowering tHcy?” Understanding folate one-carbon metabolism pathway (FOCM) genetic variation will aid us in developing new targets for therapy. The FOCM is essential in regulation of the epigenome, which controls genes in ways beyond nucleotide sequence. We present data generated from stroke-only and general populations where we identify strong association of genetic risk factors for variation in one-carbon metabolism function, characterized by the post-methionine load test. We show that *GNMT* harbors genetic and epigenetic differences that influence gene function, which may have downstream effects on the epigenome of the cell, affecting disease risk. We developed a genetic risk score that predicts post-methionine load homocysteine levels that may be useful in clinic. Finally, we identified a novel association between ischemic stroke and *ALDH1L1*, which emphasizes the clinical importance of this work. Our results highlight the importance of a concerted effort to target the FOCM (beyond tHcy) and parallel pathways in future pharmacogenetic work using the genetic variation we describe here.

## Introduction

As the fourth leading cause of death and the leading cause of disability in American adults, stroke constitutes a major public health burden. Epidemiological data consistently demonstrate an association between elevated plasma homocysteine (tHcy) and increased risk for stroke [1], cardiovascular disease [2], and dementia [3], but clinical trials of interventions to lower homocysteine have failed to demonstrate global benefit, with B12 supplementation helping to reduce risk only in subsets of the populations studied [4]–[6]. Collectively, these data support a more complicated relationship than simple biomarker and disease risk and indicate the need for new targets for risk reducing therapies. This begs the question, “Have we already missed the target of greatest clinical benefit by the time we lower homocysteine levels?” The folate one-carbon metabolism pathway (FOCM) is not only involved in the regulation of homocysteine, methionine and B-vitamin levels but also the methylation of proteins, histones, DNA and RNA. To this end, the demethylation of S-adenosyl-methionine, which gives rise to S-adenosyl-homocysteine, is the sole source of *de novo* methyl groups for the cell. Dysregulation of this step in the FOCM could have broad implications on many cellular processes, including risk for stroke and cardiovascular disease.

The post-methionine load test is a more sensitive tool for diagnosing hyperhomocysteinemia than circulating plasma homocysteine levels [7]–[10]. Additionally, “ΔPOST”, or the difference in tHcy levels before and after methionine loading in the clinic, gives a measurement of one's ability to convert methionine to homocysteine in real time and likely reflects methyl group availability in the cell. We utilized this test to analyze genetic determinants of methionine metabolism and how these differences between individuals may be functionally regulated.

Here we present a genomic, genetic and epigenetic investigation into the regulation of methionine metabolism in the Vitamin Intervention for Stroke Prevention Trial (VISP) and the Framingham Heart Study (FHS). We first present genome-wide association (GWAS) data linking five loci to differences in ability to convert methionine to homocysteine. Strikingly, all of the most significant genes identified within these loci are members of the same pathway (FOCM), a feature rarely observed in GWAS studies.

We observed haplotype differences at the *GNMT* [MIM 606628] locus, our most significant GWAS finding, and devised a scheme to test methionine loading *in vitro* based on *GNMT* genotype. Additionally, we have shown epigenetic regulation of the *GNMT* promoter, based on a CpG-SNP rs11752813, which likely contributes to *GNMT* transcription and methionine metabolism. These data may one day contribute to identification of new targets for stroke and cardiovascular disease prevention as well as other complex diseases where epigenetics play a role.

## Results

### Framingham Heart Study (FHS) and Vitamin Intervention for Stroke Prevention Trial (VISP) cohorts

The FHS cohort is a community based longitudinal study to determine the risk for cardiovascular disease and is comprised of randomly recruited participants and their family members in the town of Framingham, Massachusetts (Table 1). VISP was a multi-center, double-blind, randomized, controlled clinical trial designed to determine if vitamin supplementation reduced recurrent cerebral infarction, nonfatal myocardial infarction or mortality and is made up of unrelated individuals. The VISP cohort has a higher proportion of men when compared to the FHS, which is not surprising when considering the VISP participants have all had a stroke (Table 1). Likewise, VISP also has a greater percentage of diabetics and hypertensive individuals (Table 1). The VISP cohort consists of individuals in the top quartile of circulating tHCY levels, which was part of the recruitment requirements; whereas FHS is made up of a normal distribution of tHCY levels (Table 1). FHS participants have higher vitamin B6, B12, and folate levels on average than VISP participants. BMI and smoking status are approximately the same between VISP and FHS.

**Table 1:** Summary statistics for Framingham Heart Study (FHS) and Vitamin Intervention for Stroke Prevention (VISP) subjects.

	FHS	VISP
	N	Male(%)	Female(%)	N	Male(%)	Female(%)
Sex	3110	1461(46.98%)	1649(53.02%)	2100	1315(63.57%)	785(37.38%)

Trait	N	Mean	SD	N	Mean	SD
Age	3110	58.52	9.68	2100	67.19	10.75
tHcy (µmol/L)	3108	9.75	4.00	2090	13.32	4.83
POST tHcy (µmol/L)	2711	24.47	8.19	1947	29.71	10.11
ΔPOST tHcy (µmol/L)	2710	14.80	6.55	1944	16.50	8.81
Vitamin B_6_ (pm/mL)	3110	84.89	83.33	1897	42.45	37.38
Vitamin B_12_ (pg/mL)	3110	417.97	167.84	1954	358.79	181.91
Folate Assay with L.Casei (ng/mL)	3102	10.89	7.84	1954	25.86	15.91
BMI (kg/m^2^)	3105	27.89	5.10	1992	28.85	5.29

	N	Events(%)	N	Events(%)
Current Smoker	3107	477(15.35%)	2100	328(15.62)
Prevalent Diabetes	3110	354(11%)	2074	562(27.10)
Prevalent Hypertension (JNC-7 Stage 1 or greater)	3103	1267(40.83%)	2069	1491(71.00)
Stroke at baseline	3110	82(2.6%)		(100%) All Ischemic Stk.
ABI	3110	104 (3.3%)		
CE	3110	64 (2.1%)		
ICH	3110	28 (0.9%)		
SAH	3110	3 (0.0009%)		
OTH	3110	1 (0.0003%)		

tHCY-Circulating total plasma homocysteine. POST tHcy-Post-methionine load plasma homocysteine. ΔPOST tHcy-Difference between pre-methionine load homocysteine and post-methionine load homocysteine. BMI-Body Mass Index. Stroke subtypes: ABI-Acquired Brain Injury, CE- Cardioembolism, ICH- Intracerebral Hemorrhage, SAH-Subarachnoid Hemorrhage, OTH- stroke of other determined etiology.

The VISP study consisted of 1725 (82.1%) individuals of European descent, 258 (12.2%) individuals from African descent and 117 (5.6%) individuals of other ancestral populations. All VISP participants are unrelated. FHS samples are primarily Caucasian. In FHS the 3110 individuals contributing to GWAS belong to 1055 families with extended family size ranging from 1 to 140. In FHS, 1772 individuals have at least one blood relative in the family, 279 individuals have at least one first degree relative, 278 have at least one second degree relative, and 586 have at least one third degree relative.

### Association of Folate One-Carbon Metabolism genes with methionine metabolism

**Figure 1:**
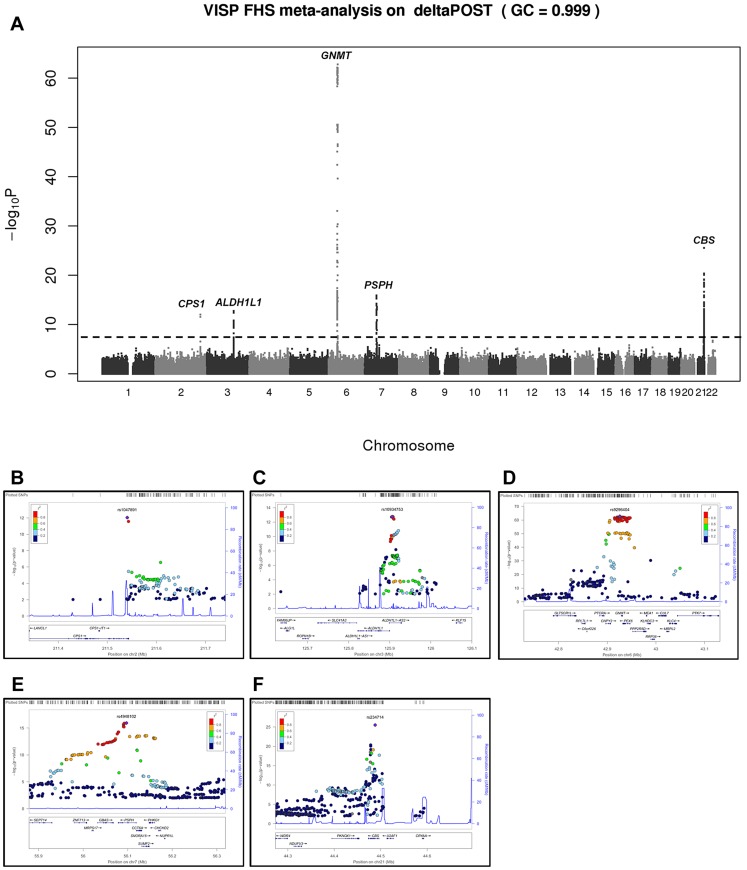
Meta-analysis and chromosome 2, 3, 6, 7 and 21 regional association of single nucleotide polymorphisms (SNPs) for ΔPOST in both VISP and FHS cohorts. A sample size-weighted meta-analysis was used. (A) Manhattan plot of meta-analysis association results for the combined VISP and FHS samples. Association p-values are noted on the Y axis (-log_10_ P value), with points above the dashed line indicating SNPs reaching or exceeding genome-wide significance (P≤5×10^−8^). (B–F) Locus Zoom plots showing the regional association of chromosomes 2, 3, 6, 7 and 21. Y-axis shows –log_10_ p-value≤0.01. X-axis shows Mb position on each chromosome. Each circle represents an independent SNP and color shading represents r^2^ values.

In a GWAS of 2,710 persons from the FHS study, five loci (*GNMT, CBS* [MIM 613381], *CPS1* [MIM 608307], *ALDH1L1* [MIM 600249] and *PSPH* [MIM 172480]) reached our pre-determined genome-wide significance threshold of 5×10^−8^ for the ΔPOST phenotype. These findings were confirmed in the VISP study sample of 2,100 persons for whom two of these loci (*GNMT* and *CBS*) independently reached genome wide significance (Figure S1, S2 and Table S1). The results of a sample size-weighted meta-analysis consisting of 4,810 subjects from both FHS and VISP confirm and strengthen the independent GWAS findings (Figure 1 and Table 2). Strikingly, all five loci identified are involved in the FOCM pathway (Figure S3). The most significant association was with *GNMT* (rs9296404, p = 1.60×10^−63^) located on 6p21.1, a region associated with large artery atherosclerotic stroke [11], [12].

**Table 2:** Most significant meta-analysis SNPs.

RS#	Chr	BP	Associated Gene	Gene Region	Alleles (Minor/Major)	MAF FHS	P-Value FHS	MAF VISP	P-Value VISP	N Meta	MAF Meta	Z-score	Direction	IQ FHS	IQ VISP	P-Value
rs9296404	6	42925803	*GNMT*	5′	C/T	0.48	1.29E-42	0.46	3.40E-23	4810	0.47	16.83	++	0.96	0.98	1.60×10^−63^
rs234714	21	44488033	*CBS*	Intron 4/5′ UTR	T/C	0.20	2.28E-18	0.22	1.03E-09	4810	0.21	10.6	++	0.49	0.98	3.15×10^−26^
rs4948102	7	56097265	*PSPH*	Intron 3	C/G	0.25	1.40E-15	0.28	5.21E-04	4810	0.26	8.29	++	0.77	0.99	1.17×10^−16^
rs10934753	3	125906179	*ALDH1L1*	5′	A/G	0.42	4.90E-13	0.40	0.0033	4810	0.42	7.37	++	0.99	Genotyped	7.3×10^−13^
rs1047891	2	211540507	*CPS1*	Ser (ACC)/Phe (AAC)	A/C	0.30	1.35E-08	0.37	1.30E-05	4810	0.32	7.14	++	0.73	Genotyped	9.10×10^−13^

RS#-SNP annotation, dbSNP build 137. Chr-Chromosome. Position from dbSNP build 137. MAF-minor allele frequency from 4810 VISP and FHS subjects used in meta-analysis. All SNPs seen here are imputed with 1000Genomes data. IQ = Imputation Quality.

### Haplotype analysis of *GNMT* locus

**Figure 2:**
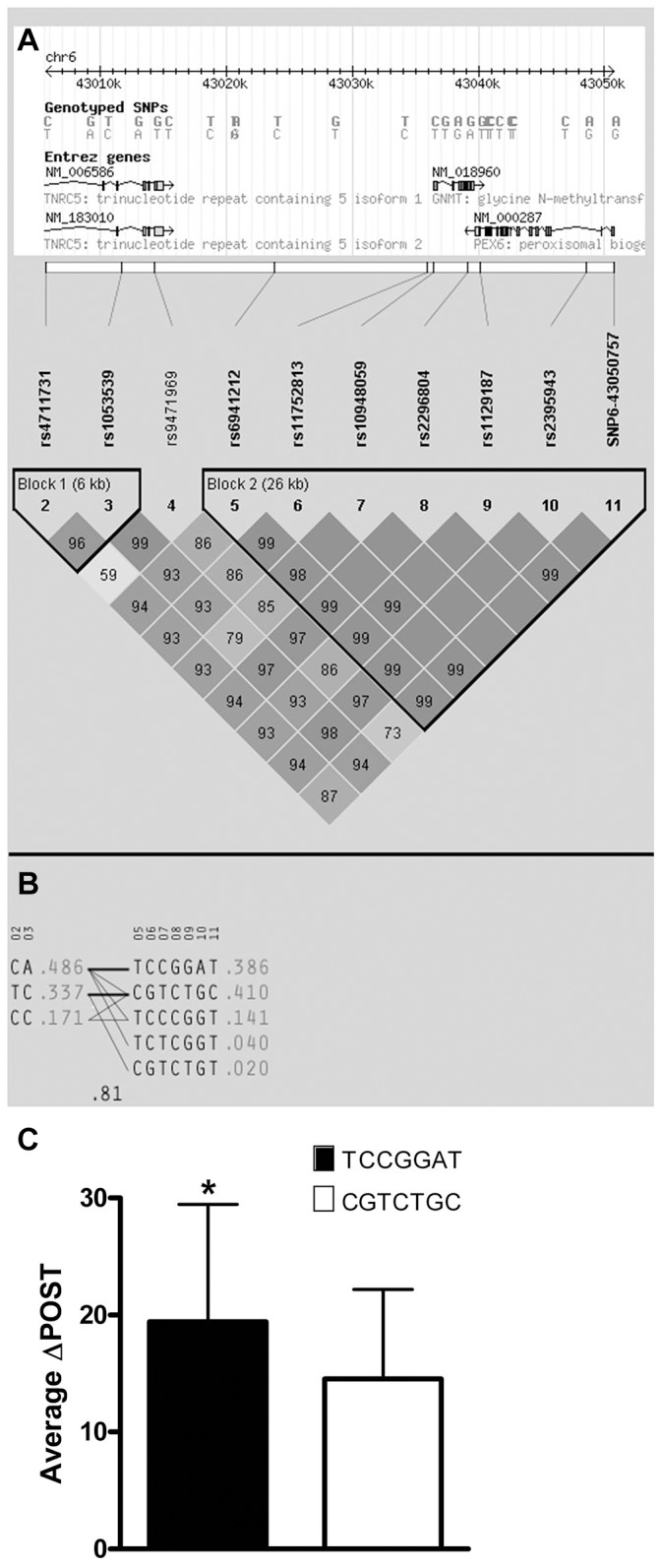
Haplotype analysis of Chr6 SNPs significantly-associated with ΔPOST in the VISP cohort. Haplotype analyses of the 10 most-associated SNPs, which are all genome wide significant, from chromosome 6, were performed using Haploview version 4.2 [13]. (A) Shows results for the chromosome 6 genomic haplotype structure in VISP, encompassing the *GNMT* gene. Genetic coordinates, Entrez gene structure, haplotype blocks and linkage disequilibrium (LD) pattern between SNPs are shown. Within LD pattern, r^2^ values are shown and are represented by shading. The darker the shading the closer the r^2^ value is to 1.0. All SNPs assessed were genotyped. (B) Shows haplotypes generated by Haploview for haplotype blocks 1 and 2. Haplotype block 2 is characterized by two major haplotypes, which account for 80% of haplotypes observed. (C) Shows the mean ΔPOST values in the VISP sample for each of the 2 major haplotype block 2 haplotypes. (_*_) p≤0.001 by student's T-test; error bars represent standard deviation from the mean (SD).

Using ten genotyped single nucleotide polymorphisms (SNPs) on chromosome 6 in the *GNMT* region, which were significantly-associated with ΔPOST in the VISP population, (Figure S1A), we conducted a haplotype analyses (Haploview software) [13]. Two major haplotypes emerged, encompassing ∼81% of the individuals in the VISP population (n = 2,100) (Figure 2A,B) and corresponding to a high methionine metabolizing haplotype (ΔPOST = 19.4 µmol/L) and a low methionine metabolizing haplotype (ΔPOST = 14.5 µmol/L) (Figure 2C). One SNP, rs10948059, which is a genotyped and located in the *GNMT* promoter, captures 100% of alleles with a mean max r^2^ of 0.722 (range 0.512–0.850). These data suggest functional differences in the *GNMT* gene impact an individual's ability to metabolize dietary methionine. The lack of a disruptive coding mutation identified by GWAS, or in sequencing of *GNMT* in 24 high and 24 low methionine metabolizers (data not shown), and the expectation that a higher rate of transcription of the *GNMT* gene should lead to higher tHcy levels, suggest a regulatory mechanism for the differences in ΔPOST rather than protein dysfunction. This metabolic difference mediated by genetic variation is of functional significance in both the general population (FHS) and a population with tHcy above the 25^th^ percentile as required by the inclusion criteria for the clinical trial (VISP).

### Promoter analysis of *GNMT*

**Figure 3:**
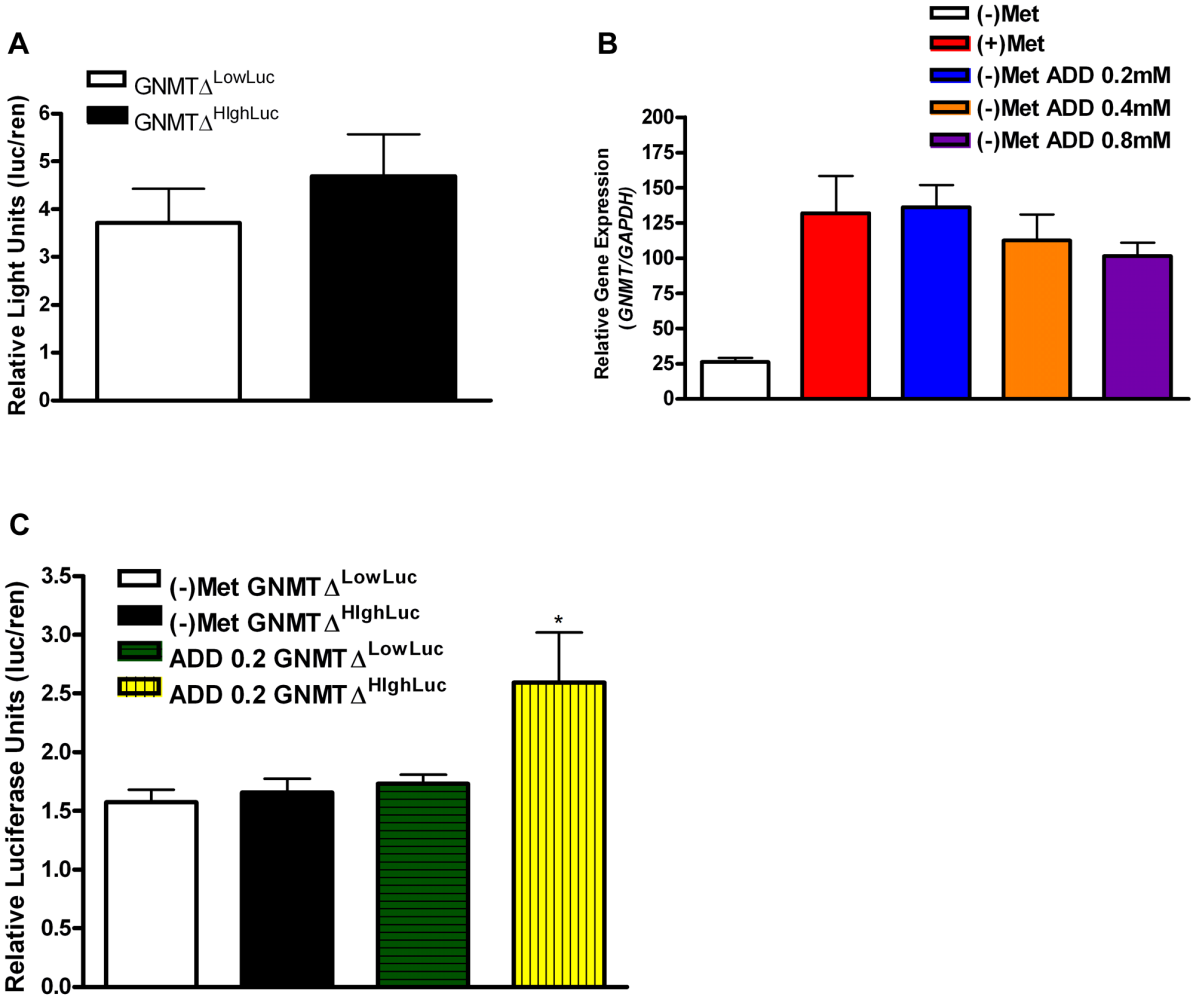
*GNMT* promoter analysis of major haplotype groups. (A) Histogram shows mean luciferase activity of the high-methionine-metabolizing haplotype (TCCGGAT) and the low-methionine-metabolizing haplotype (CGTCTGC) represented by constructs GNMT^ΔHighLuc^ and GNMT^ΔLowLuc^ cultured in standard DMEM with L-methionine (0.2 mM). (B) Shows *GNMT* qPCR analysis in HepG2 cells. Culturing conditions are as follows: (+)Met = standard complete DMEM cultured for 48 hours, (−)Met = complete DMEM without L-Methionine culture for 48 hours, (−)Met ADD 0.2 = DMEM without L-Methionine culture for 24 hours, addition of L-Methionine at 0.2 mM for 24 hours, (−)Met ADD 0.4 = DMEM without L-Methionine culture for 24 hours, addition of L-Methionine at 0.4 mM for 24 hours, (−)Met ADD 0.8 = DMEM without L-Methionine culture for 24 hours, addition of L-Methionine at 0.8 mM for 24 hours. (C) (−)Met GNMT^ΔHighLuc^ and (−)Met GNMT^ΔLowLuc^ represent HepG2 cells transfected with GNMT^ΔHighLuc^ and GNMT^ΔLowLuc^ constructs and cultured without L-methionine for 48 hours. ADD 0.2 GNMT^ΔLowLuc^ and ADD 0.2 GNMT^ΔHighLuc^ represent HepG2 cells transfected with GNMT^ΔHighLuc^ and GNMT^ΔLowLuc^ constructs cultured without L-methionine for 24 hours and with 0.2 mM L-methionine for 24 hours. * P<0.05 by students t-test. N = 3 biological replicates for all analyses. Error bars represent standard deviation from the mean (SD).

The known *GNMT* promoter [14] from the high methionine metabolizing haplotype and the low methionine metabolizing haplotype were cloned giving rise to GNMT^ΔHighLuc^ and GNMT^ΔLowLuc^ constructs (Sequence alignments in Figure S4). *GNMT* is most highly expressed in the liver; therefore HepG2 cells were used to test promoter activity in the two haplotype groups. There was a ∼30% difference in gene promoter activity between GNMT^ΔHighLuc^ and GNMT^ΔLowLuc^ constructs (Figure 3A), when cultured with L-methionine, which correlates with the differences seen between the average ΔPOST levels in our haplotype analysis (Figure 2C).

The quantitative trait, ΔPOST, is dependent on methionine dosing, therefore we starved HepG2 cells of L-methionine for 24 hours and then treated them with L-methionine for 24 hours. As seen in Figure 3C, the GNMT^ΔHighLuc^ construct responded to methionine starvation and treatment with ∼2× greater activity than the GNMT^ΔLowLuc^ construct. Above standard L-methionine culturing conditions (0.2 mM) a feedback mechanism appears to be induced, which reduces *GNMT* expression (Figure 3B). These data suggest a mechanism by which elevated levels of tHcy may arise.

### Epigenetic analysis of C/G SNP rs11752813

**Figure 4:**
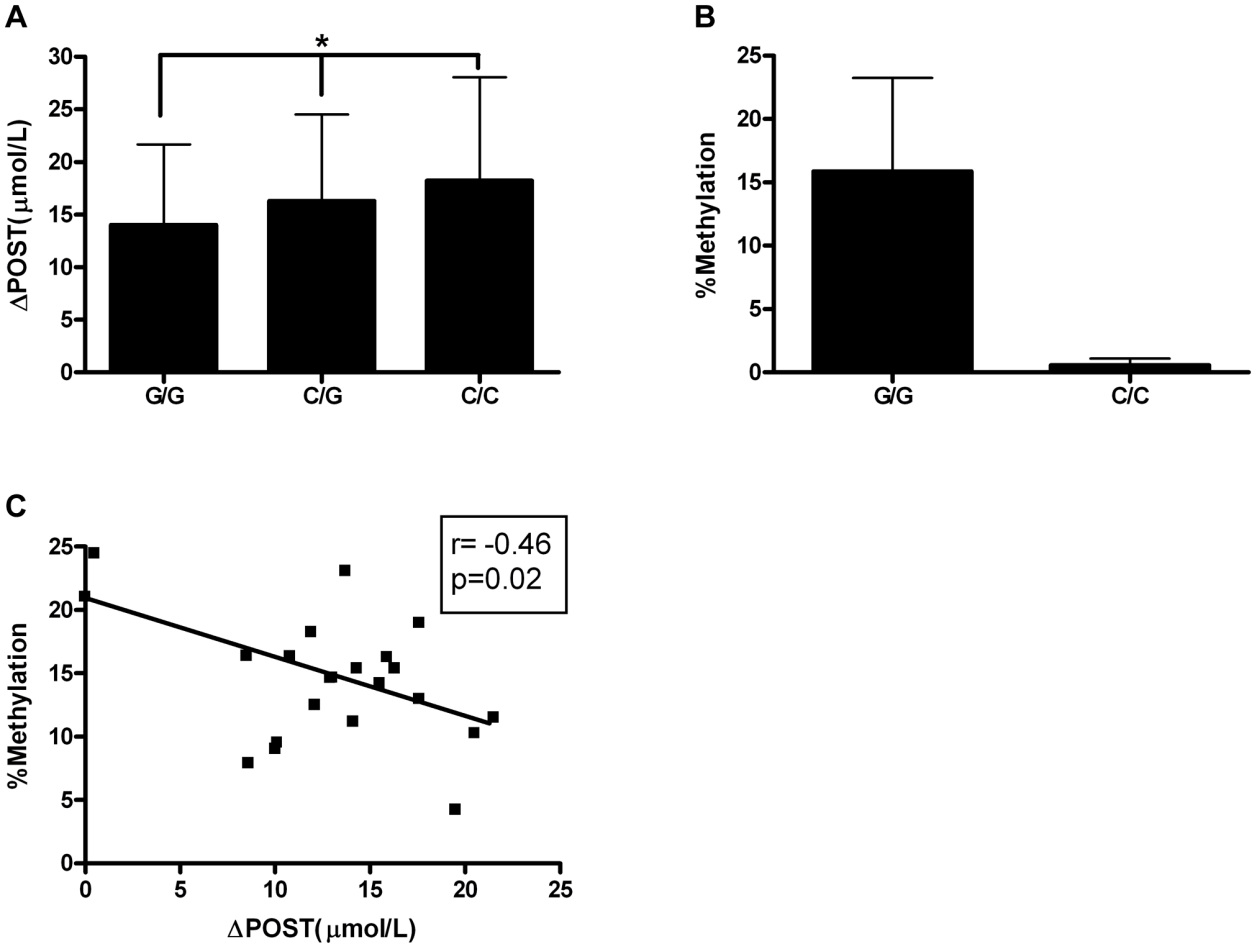
Epigenetic Evaluation of rs11752813. (A) Average ΔPOST (µmol/L) in VISP trial based on rs11752813 genotype. (*) p<0.001 by one-way ANOVA and all genotypes are significantly different by students t-test. (B) Percent methylation analyzed by bisulfite pyrosequencing of n = 23 individuals (G/G) and n = 6 individuals (C/C). (C) Linear regression analysis of percent methylation of rs11752813 based on genotype and ΔPOST (µmol/L).

Given the differences in promoter activity seen in Figure 3, we sought to identify functional variants that may play a role transcriptional activity. SNP rs11752813 was significantly associated in our meta-analysis (Figure 1A, p = 7.99×10^−32^), and either creates or eliminates a CpG site that can be methylated depending on genotype (rs11752813 and flanking sequence: C(**C/G**)A). The high methionine metabolizing haplotype (Figure 2) harbors the C/C genotype (known as GNMT^ΔHighLuc^ in Figure 3) at rs11752813, and the low methionine metabolizing haplotype (Figure 2) harbors the G/G genotype (known as GNMT^ΔLowLuc^ in Figure 3) at rs11752813. The presence of a “G” at the rs11752813 locus creates a CpG site while the presence of a “C” eliminates this CpG site. Analysis of ΔPOST values in the VISP (n = 2100) study shows that the individuals that harbor the “G” genotype at rs11752813 have significantly lower ΔPOST on average, indicating a less active *GNMT* gene (Figure 4A). Bisulfite pyrosequencing of the rs11752813 locus show that the G/G genotype can be methylated whereas the C/C genotype is not methylated (Figure 4B). These results are consistent with the central dogma of DNA methylation that only CpG sites can be methylated. Finally, 23 individuals harboring the G/G genotype at rs11752813 were bisulfite pyrosequenced and percent methylation status was plotted against individual ΔPOST values (Figure 4C). As seen in Figure 4C, even between individuals with the G/G genotype there is a strong correlation between percent methylation and ΔPOST values. These data indicate that the G/G genotype at rs11752813 creates a closed chromatin state that inhibits *GNMT* transcription and methionine metabolism.

### Genetic risk score of most significant ΔPOST variants

**Figure 5:**
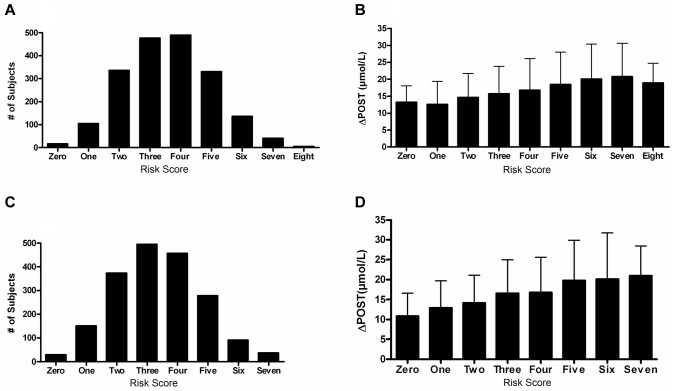
Risk score in FHS and VISP studies. (A) Distribution of risk scores among the FHS sample shows a normal distribution. Y-axis represents the number of individuals who have the given risk score seen on the x-axis. (B) X-axis represents the number of risk variants per subject in FHS. Y-axis represents the average ΔPOST value for each group containing a specific risk score. (C) Distribution of risk scores among the VISP sample shows a normal distribution. Y-axis represents the number of individuals who have the given risk score seen on the x-axis. (D) X-axis represents the number of risk variants per subject in VISP; Y-axis represents the average ΔPOST value for each group containing a specific risk score. Risk variants considered were SNPs at each of the 5 loci most significantly associated with ΔPOST in the meta-analysis. For each SNP a score of 0 was applied for homozygous non-risk variant, 1 for heterozygous at risk variant and 2 for homozygous at risk variant, derived from dosage values of 1000genomes imputation.

We next investigated cumulative effects of ΔPOST risk variants by generating a combined genetic risk score using the most significant SNPs from Table 2. Risk scores are normally distributed in both VISP and FHS (Figure 5A,C). As the risk variant load increases, the average ΔPOST levels in the VISP and FHS samples increases (Figure 5B,D). This score explains 13% of the variability of ΔPOST in FHS and 6.3% of the variability in VISP (Table 3). Because the SNPs used in this analysis are all imputed, for both the VISP and FHS studies, we repeated the analysis utilizing the most significant genotyped SNPs from each locus. This analysis yielded similar results indicating that using imputed SNPs for this risk score does not distort the analysis (Figure S5).

**Table 3:** Variance and effect size explained by risk score.

Variance Explained							
RS#	Chr	Position (bp)	Associated Gene	Gene Region	FHS V.E.	VISP V.E.	P-Value
rs9296404	6	42925803	*GNMT*	5′	0.059651	0.042	1.60×10^−63^
rs234714	21	44488033	*CBS*	Intron 4/5′ UTR	0.025319	0.017	3.15×10^−26^
rs1047891	2	211540507	*CPS1*	Ser (ACC)/Phe (AAC)	0.010857	0.0062	9.10×10^−13^
rs10934753	3	125906179	*ALDH1L1*	5′	0.01746	0.0061	7.3×10^−13^
rs4948102	7	56097265	*PSPH*	Intron 3	0.021223	0.0023	1.17×10^−16^
					Total = 0.13	Total = 0.06	

Effect Size				
	Estimate*	SE	T	P-Value
FHS	1.7(µmol/L)/risk var.	0.087	19.52	1.68×10^−79^
VISP	1.6(µmol/L)/risk var.	0.130	11.85	2.4×10^−31^

RS#-SNP annotation, dbSNP build 137. Chr-Chromosome. Position from dbSNP build 137. V.E.-Variance explained.

* = Average ΔPOST increase per risk variant added.

### *ALDH1L1* is associated with ischemic stroke in the Framingham Heart Study

Importantly, when interrogating the most significant five SNPs associated with ΔPOST, we also identified an association between the aldehyde dehydrogenase 1 family member L1 gene (*ALDH1L1*) and incident ischemic stroke in the FHS cohort (rs10934753, hazard ratio = 1.26, p = 0.015, n = 168 cases 4008 controls, analyses adjusted for age, sex and family relationships). The protein encoded by *ALDH1L1* converts 10-formyltetrahydrofolate to tetrahydrofolate and is an essential component of the FOCM pathway. These results provide a new and significant link between the FOCM pathway and risk of initial ischemic stroke. It is important to note that the VISP population consists of exclusively ischemic stroke patients examined for recurrent stroke over a 2 year period; rs10934753 was not associated with recurrent stroke. Additionally, we did not observe an association of genetic variation in *GNMT* or the other 3 loci with incident ischemic stroke in FHS but our sample has limited power to detect moderate effect size (e.g. power ranges from 20–40% to detect a hazard ratio of 1.20 for a variant with minor allele frequency ranging from 0.1–0.5).

## Discussion

Elevated tHcy has long been associated with increased risk for stroke and cardiovascular disease but to date functional evidence for the driving genetic forces behind elevated tHcy levels have only been attributable, in part, to dysfunction in the methylenetetrahydrofolate reductase gene (*MTHFR* [MIM 607093]) and *CBS* genes [15]–[18]. *MTHFR*, which also participates in the FOCM pathway, is tightly related to all the genes products identified in our study and has been implicated in susceptibility to vascular disease, neural tube defects, colon cancer and acute leukemia [19]–[24]. It is interesting to note that a prior GWAS in the individual FHS and VISP cohorts or in a meta-analysis yielded no significant results for baseline tHcy alone. This highlights the usefulness of the post-methionine load test in the diagnosis of hyperhomocysteinemia as well as the fact that we can specifically detect genetic variations that lead to differential methionine metabolism.

In the current study we followed up with a functional evaluation of our GWAS findings. These independent GWAS analyses and a meta-analysis of FHS and VISP find the *GNMT* locus as the top result. However, there were differences in the GWAS results from FHS and VISP are likely attributable to two factors: one being power (FHS consists of 610 more individuals), and two, VISP is a more homogenous population than FHS lacking a normal distribution of tHcy. Our functional studies start with the *GNMT* gene as it contributes to the majority of the variance in both VISP and FHS. The *GNMT* association makes biological sense given that GNMT catalyses the conversion of S-adenosyl-methionine (SAM) to S-adenosyl-homocysteine (SAH), using SAM as the methyl donor [25], and affects global cellular epigenetic status as the sole source of methyl groups for the cell including those used in DNA, histone, protein and RNA modifications. Additionally, it is known that global hypomethylation is seen in atherosclerosis [26], and we suspect that variation in *GNMT* could affect risk status. Further, hyperhomocysteinemia is a risk factor for stroke and cardiovascular disease, and these data indicate *GNMT* may represent a new pharmacogenetic target for reducing stroke risk. It is our assertion that targeting the FOCM pathway before methionine is converted to homocysteine may allow us to modulate parallel pathways, such as DNA and histone methylation, which directly impact stroke risk. A recent review by Krishna et al. [27] describes in detail a “tHcy memory effect” that may alter the epigenetic state of the cell and promote deleterious chances after tHcy is lowered. This further strengthens the argument to identify genetic risk, through use of our risk score, and examine parallel targets for therapy.

We did not observe any of the deleterious *GNMT* mutations associated with Glycine N-Methyltransferase Deficiency [MIM 606664] (characterized by elevated levels of plasma S-adenosylmethionine and normal plasma sarcosine) in sequencing of *GNMT* in 24 high and 24 low methionine metabolizers. Additionally, a lookup of large GWAS studies of cardiovascular and cerebrovascular disease, identified many of our most significant chromosome 6 findings in a meta-analysis for blood lipids [28], with rs2274517 being the most significant result (p = 1.37×10^−4^), suggesting that *GNMT* may play a broader role in risk traits for CVD beyond tHcy measures.

Our functional studies support the role of *GNMT* in variation of methionine metabolism. The consistent and biologically plausible results from the individual GWAS and meta-analysis emphasize that the other significant associations observed in the FOCM pathway cannot be ignored. The cystathionine-beta-synthase (*CBS*) gene has been associated with stroke [20] and methionine metabolism [29]. Additionally, *CBS* mutations are associated with homocystinuria, iridodonesis and agitated motion of the iris [MIM 236200] [30]–[33]. Within the carbamoyl phosphate synthetase one (*CPS1*) gene, ΔPOST rs1047891 was found to be associated with a missense Ser(ACC)/Phe(AAC) mutation (p = 9.10×10^−13^). These findings are related to a sex-specific association of *CPS1* with tHcy and women, performed in the Woman's Health Genome Study [34] and a Filipino population [35]. We meta-analyzed ΔPOST, rather than tHcy, and included both men (54%) and women (46%). Phosphoserine phosphatase (*PSPH*) mutations have been associated with phosphoserine phosphatase deficiency [MIM 614023], which results in pre- and postnatal growth retardation, moderate psychomotor retardation, and facial features suggestive of Williams syndrome [36], [37].

Taken together, these data present a new link to the genetics of the FOCM pathway with methionine metabolism both in stroke and non-stroke populations. Because of the impact that the FOCM pathway has on the biology of the cell, including overall epigenetic state and DNA methylation, gluconeogenesis, and DNA repair, understanding how individual genetic composition impacts this pathway is essential. The FOCM has also been implicated in many aspects of human health and the work presented may be relevant to several key biological mechanisms, affecting tumorogenesis [38], B-vitamin utilization [39], as well as cardiovascular and cerebrovascular disease risk. Additionally, it is necessary to repeat these analyses in studies of different ethnicities as both FHS and VISP are comprised of mainly individuals of European descent.

While a direct link between tHcy levels and stroke and cardiovascular disease remains debated, we have shown that understanding sequence variation in the FOCM pathway may provide a link to functional differences in the population, that in turn tie one carbon metabolism to a broad range of disease risk factors. Additionally, because tHcy-lowering therapies have had variable success in reducing stroke risk in subtype populations [6], [40], [41] and have in some cases been harmful [42], we believe that understanding how these genetic variants impact the overall FOCM and related pathways is essential to understanding the pathogenesis of stroke. Methionine metabolism provides a first clue to the impact of this pathway on cell biology.

## Materials and Methods

### Ethics statement

All human research was approved by the relevant institutional review boards, and conducted according to the Declaration of Helsinki. The Framingham Heart Study protocol was approved by the institutional review board (IRB) of the Boston University School of Medicine and all participants provided written, informed consent. The VISP study protocol was approved by the IRBs of the Wake Forest University School of Medicine (coordinating center) and University of North Carolina Chapel Hill School of Medicine (statistical center). The local IRB for each of the individual recruiting sites approved the VISP protocol and all participants provided written, informed consent. The Genomics and Randomized Trial Network (GARNET) analysis of the VISP data was approved by University of Virginia School of Medicine IRB.

### Genome wide analysis of Framingham Heart Study

FHS started in 1948 for evaluation of cardiovascular diseases and risk factors [43]. In 1971, 5124 children of the original cohort, and spouses of these children, referred to as Offspring cohort, were enrolled and have been examined approximately every four years [44]. Genotyping was performed on the Affymetrix 500K mapping array and the Affymetrix 50K supplemental array. Circulating homocysteine levels were measured on 3465 Offspring participants (N = 3464 with tHcy and N = 2999 with POST) during examination cycle 6 (1995–1998). The study sample for GWAS consists of a subset of 3110 individuals with at least one phenotype and GWAS data (N = 3108 with tHCY, N = 2711 with POST, N = 2710 with Δ POST). The sample used to examine the association between the SNPs identified in GWAS of homocysteine phenotypes and incident stroke includes 4176 original cohort and Offspring individuals (N stroke = 200 stroke; N ischemic stroke = 168).

### Homocysteine measurement and stroke classification

Plasma tHcy levels were measured using high-performance liquid chromatography with fluorescence detection [45].

Clinical stroke was defined as rapidly developing signs of focal neurologic disturbance of presumed vascular etiology lasting more than 24 hours. Additional details of stroke classification and diagnosis can be found in prior publications [45]–[48].

### Imputation and statistical analyses

Imputation of about 11 million 1000 Genomes SNPs (1000G Phase I Integrated Release Version 3 Haplotypes: 2010–11 data freeze, 2012-03-14 haplotypes) was performed using MACH version 1.0.16 (http://www.sph.umich.edu/csg/abecasis/MACH/) based on 412,053 good quality SNPs (excluded SNPs were characterized by call rate <97%, pHWE<1E-6, Mishap p<1e-9, >100 Mendel errors, MAF<1%).

Prior to association analysis, homocysteine phenotypes were normalized by replacing its observed value with the corresponding quantile under normal distribution. For GWAS, linear mixed effects models were fitted with the transformed phenotypes as dependent variables, individual SNP genotype as a fixed effect, and person specific random effects with correlation coefficient between two individuals being twice their kinship coefficient to account for correlation within extended families [49]. FHS GWAS has adjusted for age, sex and first 10 eigenstrat principal components in the linear mixed effects mode (Table S2). Cox proportional hazard model with a robust variance to account for familial relationship was fitted to relate SNPs identified in GWAS with stroke outcomes [50].

### VISP Imputation

Imputation was performed using all SNPs and samples passing basic quality filters. In brief, SNPs were selected using the recommended composite quality filter that emerged from the genotype data cleaning process. Samples were selected to have an overall missing call rate <2%, while certain sample-chromosome combinations were also excluded where a gross chromosomal anomaly was detected or when the chromosome-specific missing call rate was >5%. These study data were imputed to a phase 1 interim release from the 1000 Genomes (1000G) Project [51]. Imputation target variants were defined as those with MAF≥0.005 across all 629 1000G samples.

Imputation was carried out using BEAGLE imputation software [52] (v3.3.1) for chromosomes 1–22 and the X chromosome. The imputed dataset contained total 7,500,450 variants; 766,577 of which (10.2%) were observed from the array genotyping. In addition to the primary imputation analysis, additional imputations were run on chromosome 22 and the X chromosome, masking a random 10% of observed SNPs to empirically assess imputation quality. The squared correlation between observed and imputed allelic dosages (dosage r^2^) was used to summarize the imputation quality. The median dosage r^2^ was 0.933 for chromosome 22 masked SNPs and 0.930 for X chromosome masked SNPs. The imputed dataset, along with a detailed report on imputation methodology, is available through the authorized access portion of the VISP dbGaP posting.

Genotyping was performed on the Illumina HumanOmni1-Quad-v1 array (Illumina, Inc.) at the Center for Inherited Disease Research, Johns Hopkins University. The genome-wide association analysis was conducted using PLINK v1.0.7. Multivariate linear regression model was used to test correlation of quantitative traits and SNP markers. Using the KING software, the top 10 principle components were derived from genotype data [53] and subsequently used to adjust for population heterogeneity, in addition, age and gender were also included as covariates in the model. The VISP population consists of genetically confirmed unrelated individuals and no adjustments were made to the analysis for relatedness. To normalize phenotypic traits, inverse normal transformation was applied to values of POST and ΔPOST. The same regression model was employed to perform association tests between the phenotype and expected allele counts.

### Meta-analysis of Δ post methionine load test homocysteine levels

Meta-analysis of the 2100 VISP and 3110 FHS cohorts was conducted using the METAL software [54]. The sample size of each study was used as weight, and the sign of the beta value of each SNP coded allele was used as the direction for association (Table S3).

### Regional association plots

Figure 1 regional association plots were created using the locus zoom “plot your own data” function (https://statgen.sph.umich.edu/locuszoom/genform.php?type=yourdata). Plots were created utilizing the genome build/LD population hg19/1000 Genomes Mar 2012 EUR.

### Cell culture

Unless otherwise noted, HepG2 cell lines were cultured in complete media containing high glucose Dulbecco's Modified Eagle's Medium (DMEM) (Invitrogen) with 10% (v/v) FBS, 2 mM L-glutamine supplemented with 1× nonessential amino acids, 1 mM sodium pyruvate (Invitrogen) and 1X antibiotic-antimycotic (Invitrogen). Cells were maintained at 37°C in a 5% CO_2_ incubator.

### In vitro L-methionine treatment

HepG2 cells were culture in complete media as described above for 24 hrs in 25 cm^2^ tissue culture flasks or 6 well culture plates. For methionine starvation, after 24 hrs media was removed and replaced with either complete media or complete media lacking L-methionine. After 24 hrs, cells starved of methionine were supplemented with L-methionine (Ameresco) at concentrations of (0.2 mM, 0.4 mM, 0.8 mM).

### Real time qPCR

RNA isolation was conducted using the Qiagen RNeasy kit according to standard manufacture's protocols. cDNA synthesis was conducted using 1 ug of total RNA and the Verso cDNA Synthesis Kit (Fisher Scientific). A 3∶1 mix (v/v) of random hexamers and anchored oligo-dT was used following standard theromocycling conditions. For *GNMT* quantitative real-time PCR, Taqman MGB probes and primers were used (Hs002219089) (Applied Biosystems). All samples were run in triplicate in 10 µl reaction volumes. PCR conditions were the default settings of the ABI Prism 7900 HT Sequence Detection System (Applied Biosystems) using the standard curve setting to achieve raw data, which was analyzed in Microsoft Excel. The cycle threshold (Ct) was determined during the geometric phase of the PCR amplification plots as automatically set by the 7900 software. Relative differences in transcript levels were quantified using the ΔΔCt method with *GAPDH* [MIM 138400] (probe 4333764F) mRNA as an endogenous control.

### Creation of luciferase plasmids

*GNMT* promoters were PCR amplified using primers (Forward: 5′-CGGGGTACCACAGAGCGAGACTGTGTC-3′, Reverse: 5′-GCGAGATCTCCTGCGCCGCGCCTGGCT-3′) as previously described [14]. One VISP case was chosen for TA cloning from the high ΔPOST and low haplotype ΔPOST haplotypes. Promoters were cloned into the StrataClone PCR Cloning vector according to the standard protocols (Agilent Technologies). Promoters were next restriction digested and ligated into the pGL3 luciferase plasmid (Promega) using KpnI and SacI enzymes (New England Biolabs) giving rise to GNMT^ΔHighLuc^ and GNMT^ΔLowLuc^.

### Haplotype analysis

Haploview version 4.2 [13] was used to analyze haplotype blocks on chromosome 6 from the VISP population. 10 SNPs with p≤5×10^−8^ were assessed using version 3, release 27, analysis panel CEU+TSI.

Average ΔPOST values were taken from the VISP population, and any individuals with missing data from 1 or more SNPs were excluded from the analysis. The top two haplotypes, encompassing 80% of the total VISP population were assessed.

### GNMT promoter analysis

2×10^4^ HepG2 cells were transfected in quadruplicate with GNMT^ΔHighLuc^ or GNMT^ΔLowLuc^ and pGL4.74[hRluc/TK] (Promega) at a 10∶1 ratio using TransIt-LT1 transfection reagent (Mirus biosciences) in 96 well plates. Luciferase assays were conducted following the Dual-glo kit standard protocol (Promega). Luciferase readings were taken using the Beckman Coulter DTX880 luminometer at a 1 second integration time. Firefly luciferase measures from GNMT^ΔHighLuc^ or GNMT^ΔLowLuc^ were taken for each well, followed by treatment for renilla luciferase activity and renilla measurement. Relative luciferase activity of each promoter was calculated by dividing the average firefly luciferase counts from GNMT^ΔHighLuc^ or GNMT^ΔLowLuc^ by pGL4.74[hRluc/TK] for each independent condition. L-Methionine treatment used 0.2 mM reagent. Total relative luciferase activity for each plasmid encompasses the average of 3 biological replicates.

### Pyrosequencing

Pyrosequencing was performed as previously described [55]. Primers are as follows, Forward: AGTAGAGAAGTGTTAGTTAGGTTTTAT, Reverse (Biotin labeled): ACCCATACAAAAAAAACAAAAAAAATCTC, Sequencing primer: TTTGGATTAGGTGGATAG.

### Risk score analysis

Scores were determined by using imputation dosage measures from VISP and FHS. Alleles were assessed for the average ΔPOST values in FHS and VISP. If a dosage for a homozygous SNP was associated with high homocysteine on average (i.e. close to the value 2) the number was not changed. However, if the homozygous allele was not associated with the risk variant (i.e. close to 0) but was represented as a number above 1.5, 2 was subtracted from that number and made positive. After correction, homozygous risk variants would have a dosage value near 2, heterozygous variants would have a value near 1, and non risk variants would be assigned a number near 0. All imputation dosage values were summed. The sum of each risk value was then taken for each individual to give a score from 0 to a possible 10, and that score was rounded to the nearest integer.

For calculation of the variance explained by the risk score, linear regression was used for VISP, and a linear mixed effects model was used for FHS, as these methods were used in the initial GWAS for each study.

### Statistics

All statistics for Figures 2–4 were performed using GraphPad Prism 4. Tests used are indicated in Figure legends. Significance threshold was set at p = 0.05.

## Supporting Information

Figure S1 Association of single nucleotide polymorphisms (SNPs) with ΔPOST. In Panels A and B, each SNP is represented by a point. The higher the point, the lower the negative -log10 p-value seen on the y-axis and the more significant the association with ΔPOST. Points above the dashed line indicate SNPs with a p-value of less than 5×10^−8^. (A) GWAS of the VISP cohort for ΔPOST imputed with 1000 Genomes. (B) GWAS of the FHS cohort for ΔPOST using imputation with 1000 Genomes.
(PDF)

Figure S2 QQ plots of meta-analyzed data of ΔPOST. Minus logarithm to base 10 of the p-values are plotted against the minus logarithm to base 10 of the quantiles of uniform (0,1) distribution to compare the p-value distribution with expected uniform distribution with all SNPs with 1 Mb of the top SNP of each associated loci removed. A diagonal line was drawn to show any departure of the p-value distribution from expected uniform distribution. The plotted genomic control parameter (lambda) is the ratio of median chi-squared test statistics to the median of an expected 1 degree-of-freedom chi-squared distribution. (A) All data from meta-analysis. (B) All data excluding meta-analysis significant SNPs and all SNPs within 1MB to rule out LD.
(PDF)

Figure S3 Folate one-carbon metabolism pathway. Diagram shows all genome wide significant genes and their role in FOCM.
(PDF)

Figure S4 Alignment of GNMT promoters from HighDeltaPOST and LowDeltaPOST constructs. Sequences were aligned using Bioedit software (http://www.mbio.ncsu.edu/bioedit/bioedit.html) and the pairwise alignment function.
(PDF)

Figure S5 Genetic risk score in VISP using top genotyped SNPs. (A) Distribution of genetic risk scores in VISP. (B) Risk score vs. ΔPOST in VISP. Error bars represent standard error.
(PDF)

Table S1 Independent GWAS results for ΔPOST phenotype in VISP and FHS.
(PDF)

Table S2 Correlation coefficients of principal components and ΔPOST. As an exploratory analysis, we calculated the Pearson correlation coefficients of ΔPost with all the covariates (age, first 10 principal components from EIGENSTRAT analysis (FHS) or KING (VISP)) and corresponding p-values in FHS and VISP.
(PDF)

Table S3 Test of heterogeneity for VISP-FHS meta-analysis of ΔPOST. We use Cochrane's Q statistic to test heterogeneity between FHS and VISP results. Cochrane's Q statistic is the sum of squared deviations of each study's effect estimate from the overall meta-analyzed effect estimate, weighted by inverse variance of the corresponding estimates.
(XLSX)
