# Correction: Genome-Wide Meta-Analysis of Homocysteine and Methionine Metabolism Identifies Five One Carbon Metabolism Loci and a Novel Association of ALDH1L1 with Ischemic Stroke

**DOI:** 10.1371/journal.pgen.1004571

**Published:** 2014-07-18

**Authors:** The PLOS Genetics Staff

Two authors are not included in the author byline.

Jessica J. Connelly should be listed as the eighth author and affiliated with Robert M. Berne Cardiovascular Research Center, University of Virginia, Charlottesville, Virginia, United States of America, and Department of Medicine, Division of Cardiovascular Medicine, University of Virginia, Charlottesville, Virginia, United States of America. The contributions of this author are as follows: Conceived and designed the experiments and Edited the Manuscript.

Travis S. Lillard should be listed as the ninth author and affiliated with Department of Medicine, Division of Cardiovascular Medicine, University of Virginia, Charlottesville, Virginia, United States of America. The contributions of this author are as follows: Conceived and designed the experiments and Edited the Manuscript.

Affiliation 2 and 9 also have information missing and are listed correctly below. The fully corrected author and affiliation list and author contribution list is provided below.

Stephen R. Williams,^#1,2^ Qiong Yang,^#3,4^ Fang Chen,^1^ Xuan Liu,^3^ Keith L. Keene,^1,5,6,7^ Paul Jacques,^8^ Wei-Min Chen,^1^ Jessica J. Connelly^2,9^, Travis S. Lillard^9^, Galit Weinstein,^10^ Fang-Chi Hsu,^11^ Alexa Beiser,^3,10^ Liewei Wang,^12^ Ebony Bookman,^13^ Kimberly F. Doheny,^14^ Philip A. Wolf,^10^ Michelle Zilka,^14^ Jacob Selhub,^8^ Sarah Nelson,^15^ Stephanie M. Gogarten,^15^ Bradford B. Worrall,^5,16,¶^ Sudha Seshadri,^10,¶^ Michèle M. Sale,^1,2,17,¶*^ the Genomics and Randomized Trials Network,^‡^ and the Framingham Heart Study^‡^

^1^Center for Public Health Genomics, University of Virginia, Charlottesville, Virginia, United States of America

^2^Robert M. Berne Cardiovascular Research Center, University of Virginia, Charlottesville, Virginia, United States of America

^3^Department of Biostatistics, Boston University School of Public Health, Boston, Massachusetts, United States of America

^4^The Framingham Heart Study, Framingham, Massachusetts, United States of America

^5^Department of Public Health Sciences, University of Virginia, Charlottesville, Virginia, United States of America

^6^Department of Biology, East Carolina University, Greenville, North Carolina, United States of America

^7^Center for Health Disparities Research, East Carolina University, Greenville, North Carolina, United States of America

^8^Jean Mayer USDA Human Nutrition Research Center on Aging and Friedman School of Nutrition Science and Policy, Tufts University, Boston, Massachusetts, United States of America

^9^Department of Medicine, Division of Cardiovascular Medicine, University of Virginia, Charlottesville, Virginia, United States of America

^10^Department of Neurology, Boston University School of Medicine, Boston, Massachusetts, United States of America

^11^Department of Biostatistical Sciences, Wake Forest School of Medicine, Winston-Salem, North Carolina, United States of America

^12^Department of Molecular Pharmacology and Experimental Therapeutics, Mayo Clinic College of Medicine, Rochester, Minnesota, United States of America

^13^National Human Genome Research Institute, Bethesda, Maryland, United States of America

^14^Center for Inherited Disease Research, Johns Hopkins University, Baltimore, Maryland, United States of America

^15^Department of Biostatistics, University of Washington, Seattle, Washington, United States of America

^16^Department of Neurology University of Virginia, Charlottesville, Virginia, United States of America

^17^Department of Biochemistry and Molecular Genetics, University of Virginia, Charlottesville, Virginia, United States of America

^#^Contributed equally.

¶ BBW, SS, and MMS are joint senior authors on this work.

‡ Membership of the Genomics and Randomized Trials Network and the Framingham Heart Study is provided in the Acknowledgments.

* E-mail: ms5fe@eservices.virginia.edu

The authors have declared that no competing interests exist.

Conceived and designed the experiments: SRW QY FC XL KLK PJ WMC JJC TSL GW FCH AB LW EB KFD PAW MZ JS SN SMG SS MMS BBW. Performed the experiments: SRW FC QY XL KFD SN WMC. Analyzed the data: SRW FC QY XL FCH WMC KLK SS BBW MMS. Wrote the paper: SRW QY SS MMS BBW. Edited the manuscript: SRW QY FC XL KLK PJ WMC JJC TSL GW FCH AB LW EB KFD PAW MZ JS SN SMG SS MMS BBW.
