# Supplementary figures and images for: Genome-Wide Meta-Analysis of Homocysteine and Methionine Metabolism Identifies Five One Carbon Metabolism Loci and a Novel Association of ALDH1L1 with Ischemic Stroke
Source: PLoS Genet. 2014 Mar 20;10(3):e1004214. doi: 10.1371/journal.pgen.1004214 (PMC3961178; doi:10.1371/journal.pgen.1004214)

**A**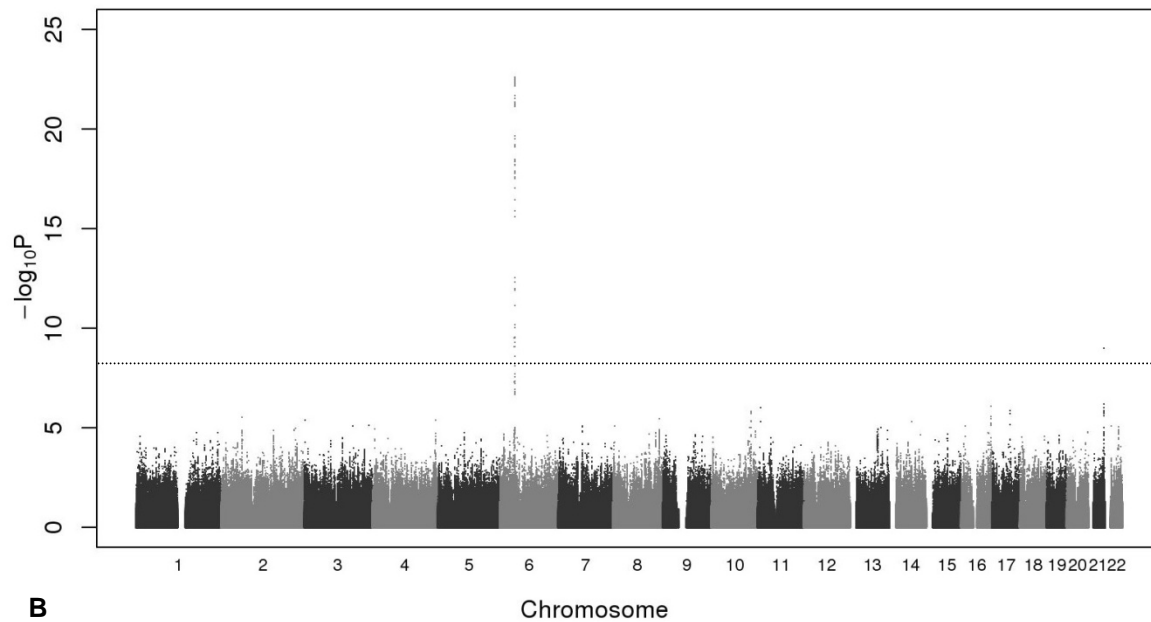**B**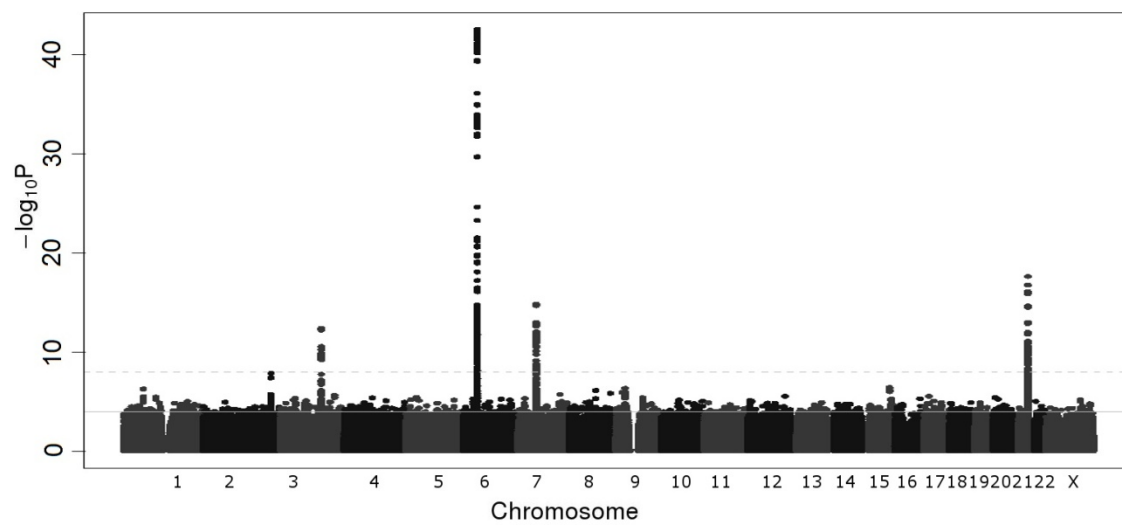

Supplement: Figure S1 — Association of single nucleotide polymorphisms (SNPs) with ΔPOST. In Panels A and B, each SNP is represented by a point. The higher the point, the lower the negative -log10 p-value seen on the y-axis and the more significant the association with ΔPOST. Points above the dashed line indicate SNPs with a p-value of less than 5×10^−8^. (A) GWAS of the VISP cohort for ΔPOST imputed with 1000 Genomes. (B) GWAS of the FHS cohort for ΔPOST using imputation with 1000 Genomes. (PDF) [file pgen.1004214.s001.pdf]

**A**

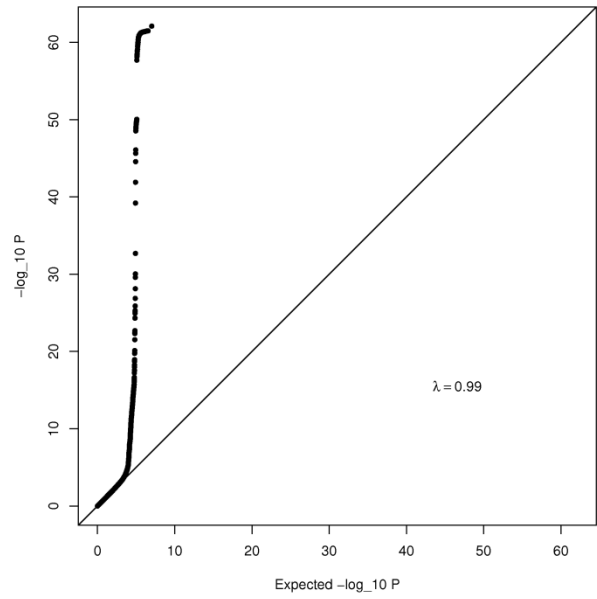

**B**

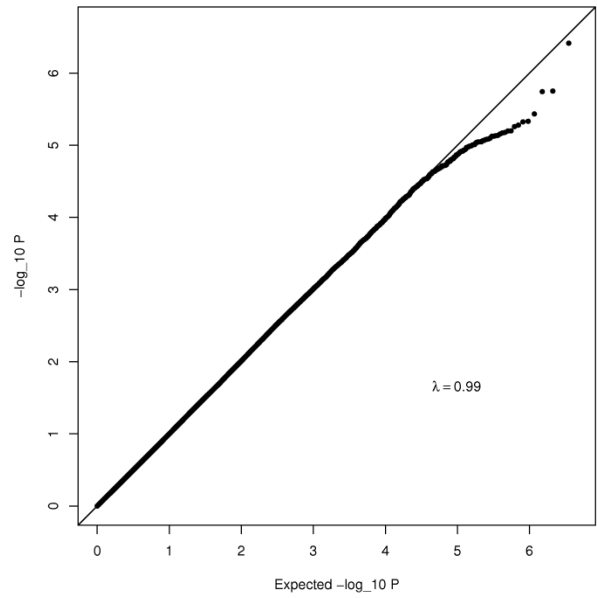

Supplement: Figure S2 — QQ plots of meta-analyzed data of ΔPOST. Minus logarithm to base 10 of the p-values are plotted against the minus logarithm to base 10 of the quantiles of uniform (0,1) distribution to compare the p-value distribution with expected uniform distribution with all SNPs with 1 Mb of the top SNP of each associated loci removed. A diagonal line was drawn to show any departure of the p-value distribution from expected uniform distribution. The plotted genomic control parameter (lambda) is the ratio of median chi-squared test statistics to the median of an expected 1 degree-of-freedom chi-squared distribution. (A) All data from meta-analysis. (B) All data excluding meta-analysis significant SNPs and all SNPs within 1MB to rule out LD. (PDF) [file pgen.1004214.s002.pdf]

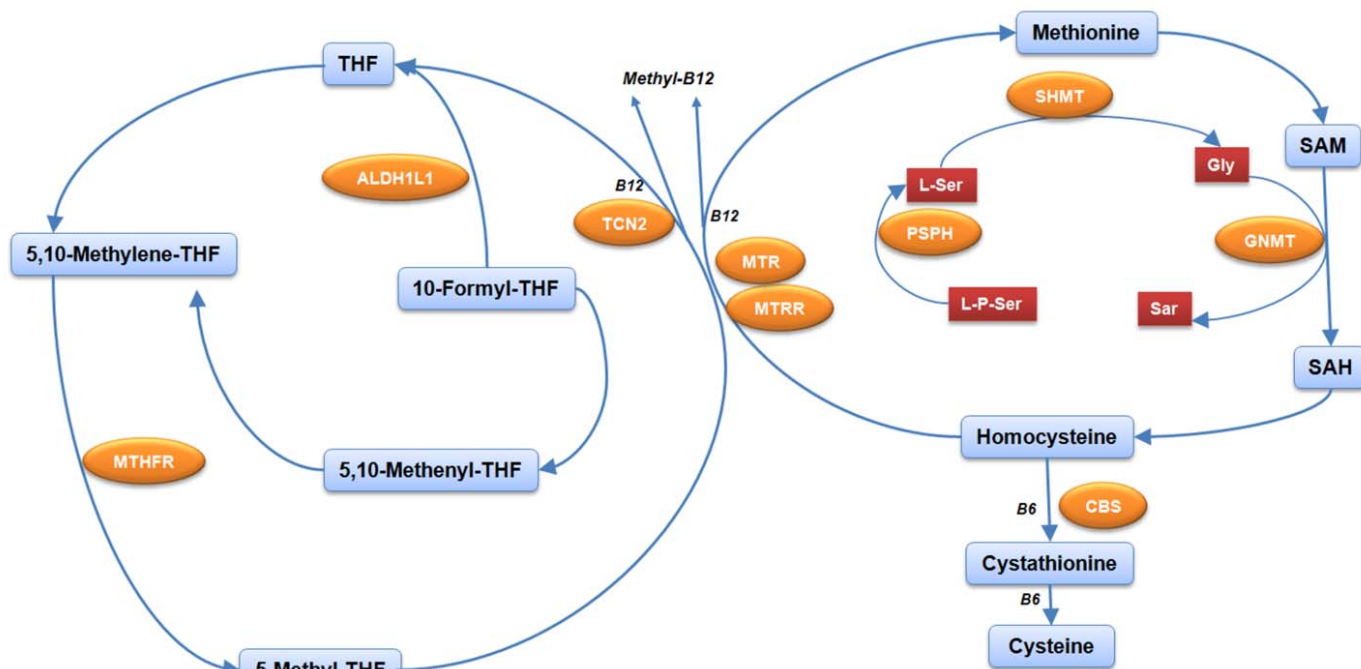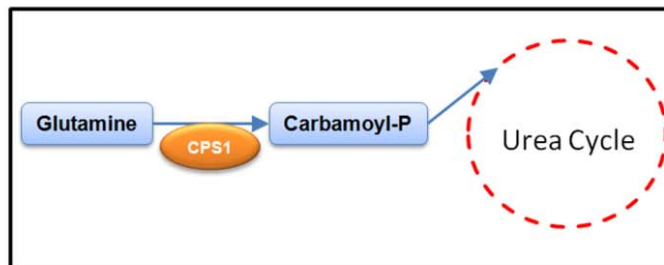

Supplement: Figure S3 — Folate one-carbon metabolism pathway. Diagram shows all genome wide significant genes and their role in FOCM. (PDF) [file pgen.1004214.s003.pdf]

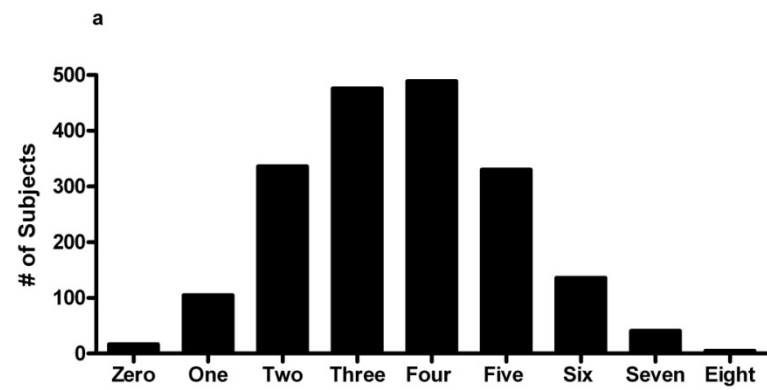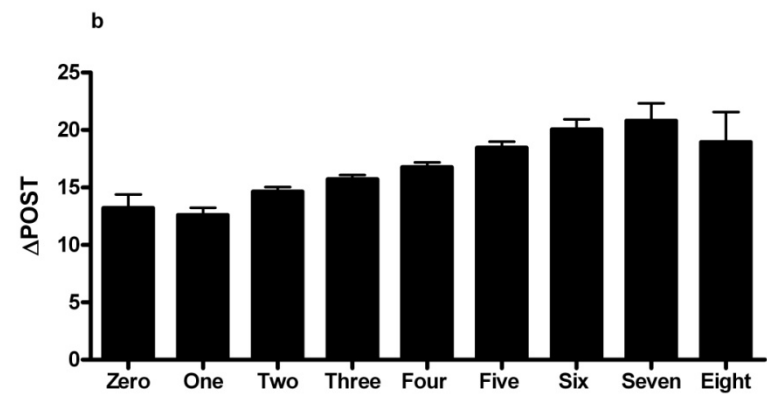

Supplement: Figure S5 — Genetic risk score in VISP using top genotyped SNPs. (A) Distribution of genetic risk scores in VISP. (B) Risk score vs. ΔPOST in VISP. Error bars represent standard error. (PDF) [file pgen.1004214.s005.pdf]
