# Supplementary material for: Genome-Wide Meta-Analysis of Homocysteine and Methionine Metabolism Identifies Five One Carbon Metabolism Loci and a Novel Association of ALDH1L1 with Ischemic Stroke
Source: PLoS Genet. 2014 Mar 20;10(3):e1004214. doi: 10.1371/journal.pgen.1004214 (PMC3961178; doi:10.1371/journal.pgen.1004214)
Supplement: Figure S4 — Alignment of GNMT promoters from HighDeltaPOST and LowDeltaPOST constructs. Sequences were aligned using Bioedit software (http://www.mbio.ncsu.edu/bioedit/bioedit.html) and the pairwise alignment function. (PDF) [file pgen.1004214.s004.pdf]

```
                                  10        20        30        40        50
                          ....|....|....|....|....|....|....|....|....|....|
Reference          1      AGCCTGGCGACAGAGCGAGACTGTGTCTCAAAACAAAAACAAAAACAAAA
HighDeltaPOST      1      -GGGTACC-ACAGAGCGAGACTGTGTCTCAAAACAAAAACAAAAACAAAA
LowDeltaPOST       1      -GGGTACC-ACAGAGCGAGACTGTGTCTCAAAACAAAAACAAAAACAAAA

                                  60        70        80        90       100
                          ....|....|....|....|....|....|....|....|....|....|
Reference          51     AAAAACCCTGCAGTTGCTTCCATGTTTAGGCGCTGCCCACCAAGGGTCCC
HighDeltaPOST      49     AAAAACCCTGCAGTTGCTTCCATGTTTAGGCGCTGCCCACCAAGGGTCCC
LowDeltaPOST       49     AAAAACCCTGCAGTTGCTTCCATGTTTAGGCGCTGCCCACCAAGGGTCCC

                                 110       120       130       140       150
                          ....|....|....|....|....|....|....|....|....|....|
Reference          101    AAGTTGGAAAGGAAGGAGGAGAGAGGCAGAGAGAGAGAGAGAGAGAGAGA
HighDeltaPOST      99     AAGTTGGAAAGGAAGGAGGAGAGAGGCAGAGAGAGAGAGAGA--------
LowDeltaPOST       99     AAGTTGGAAAGGAAGGAGGAGAGAGGCAGAGAGAGAGAGAGAGAGAGAGA

                                 160       170       180       190       200
                          ....|....|....|....|....|....|....|....|....|....|
Reference          151    GA--GAGAGAGAAGCGCGAGAGCGGCCCTGTAATTGAGCAGAAAGGAAAG
HighDeltaPOST      140    ----GAGAGAGAAGCGCGAGAGCGGCCCTGTAATTGAGCAGAAAGGAAAG
LowDeltaPOST       149    GAGAGAGAGAGAAGCGCGAGAGCGGCCCTGTAATTGAGCAGAAAGGAAAG

                                 210       220       230       240       250
                          ....|....|....|....|....|....|....|....|....|....|
Reference          199    GGAGAAAAATGAATCCCAAACTTTGGGCTTACCTCTTTCTGCTGGCTGGC
HighDeltaPOST      187    GGAGAAAAATGAATCCCAAACTTTGGGCTTACCTCTTTCTGCTGGCTGGC
LowDeltaPOST       199    GGAGAAAAATGAATCCCAAACTTTGGGCTTACCTCTTTCTGCTGGCTGGC

                                 260       270       280       290       300
                          ....|....|....|....|....|....|....|....|....|....|
Reference          249    TCGCCAAAATATGTTAACGGTAGAGGATGTCCAGGTTCTTGGCATCTTGA
HighDeltaPOST      237    TCGCCAAAATATGTTAACGGTAGAGGGTGTCCAGGTTCTTGGCATCTTGA
LowDeltaPOST       249    TCGCCAAAATATGTTAACGGTAGAGGATGTCCAGGTTCTTGGCATCTTGA

                                 310       320       330       340       350
                          ....|....|....|....|....|....|....|....|....|....|
Reference          299    ACAAACAATTGGACAAAATGCACAAACAAAGCAAGAAAGGAATGAAGGAT
HighDeltaPOST      287    ACAAACAATTGGACAAAATGCACAAACAAAGCAAGAAAGGAATGAAGGAT
LowDeltaPOST       299    ACAAACAATTGGACAAAATGCACAAACAAAGCAAGAAAGGAATGAAGGAT

                                 360       370       380       390       400
                          ....|....|....|....|....|....|....|....|....|....|
Reference          349    TTTATTGAAAATGAAAGTACACTCCACAGTGTGGGAGCGAGCATGAGCAT
HighDeltaPOST      337    TTTATTGAAAATGAAAGTACACTCCACAGTGTGGGAGCGAGCATGAGCAT
LowDeltaPOST       349    TTTATTGAAAATGAAAGTACACCCCCCAGTGTGGGAGCGAGCATGAGCAT

                                 410       420       430       440       450
                          ....|....|....|....|....|....|....|....|....|....|
Reference          399    AGGGGCTCAAAGGCCCTGTTACA----TTTTGTGAGTTTAAATACCCTCT
HighDeltaPOST      387    AGGGGCTCAAAGGCCCTGTTACAGAGTTTTTGTGAGTTTAAATACCCTCT
LowDeltaPOST       399    AGGGGCTCAAAGGCCCTGTTACA----TTTTGTGAGTTTAAATACCCTCT

                                 460       470       480       490       500
                          ....|....|....|....|....|....|....|....|....|....|
Reference          445    ACTTGGGGTACACCCTATGTAAATGAAGAGGATGAAGTAAAGTTACAGTT
```

```
HighDeltaPOST   437  ACTTGGGGTACACCCTATGTAAATGAAGAGGATGAAGTAAAGTTACAGTT
LowDeltaPOST    445  ACTTGGGGTACACCCTATGTAAATGAAGAGGATGAAGTAAAGTTACAGTT

                               510       520       530       540       550
                     ....|....|....|....|....|....|....|....|....|....|
Reference       495  ACAAAGTTATTAATGGCGGGCATACGCCCTATGGAGAGGATATTTCCCGT
HighDeltaPOST   487  ACAAAGTTATTAATGGCGGGCATACGCCCTATGGAGAGGATATTCCCCGT
LowDeltaPOST    495  ACAAAGTTATTAATGGCGGGCATACGCCCTATGGAGAGGATATTTCCCGT

                               560       570       580       590       600
                     ....|....|....|....|....|....|....|....|....|....|
Reference       545  TATAACTGAAGTGTGAATTGGCCTTATGTTCCCTGTCTCTAGACCCTATT
HighDeltaPOST   537  TACAACTGAAGTGTGAATTGGCCTTATGTTCCCTGTCTCTAGACCCTATT
LowDeltaPOST    545  TATAACTGAAGTGTGAATTGGCCTTATGTTCCCTGTCTCTAGACCCTATT

                               610       620       630       640       650
                     ....|....|....|....|....|....|....|....|....|....|
Reference       595  CTCCTGCTTCCTGTGAAAGCAGAGGGTTTTGTTTGTTTTTTGCTTTTTGT
HighDeltaPOST   587  CTCCTGCTTCCTGTGAAAGCAGAGGGTTTTGTTTGTTTTTTGCTTTTTGT
LowDeltaPOST    595  CTCCTGCTTCCTGTGAAAGCAGAGGGTTTTGTTTGTTTTTTGCTTTTTGT

                               660       670       680       690       700
                     ....|....|....|....|....|....|....|....|....|....|
Reference       645  TTTTTGAGACGGAGTCTTGCTCTGTTGCCCAGGCTGGAGTGCAGTGGTGC
HighDeltaPOST   637  TTTTTGAGACGGAGTCTTGCTCTGTTGCCCAGGCTGGAGTGCAGTGGTGC
LowDeltaPOST    645  TTTTTGAGACGGAGTCTTGCTCTGTTGCCCAGGCTGGAGTGCAGTGGTGC

                               710       720       730       740       750
                     ....|....|....|....|....|....|....|....|....|....|
Reference       695  ACTCGGCTCACTGCAACTTCCGCCTTCCCGGGTTCAAGCGATTCTCCTGG
HighDeltaPOST   687  ACTCGGCTCACTGCAACTTCCGCCTTCCCGGGTTCAAGCGATTCTCCTGG
LowDeltaPOST    695  ACTCGGCTCACTGCAACTTCCGCCTTCCCGGGTTCAAGCGATTCTCCTGG

                               760       770       780       790       800
                     ....|....|....|....|....|....|....|....|....|....|
Reference       745  CTCAGCCTCCGGAGTAGCTAGGACTACAGGCGCGTGTCACCATGTCCCAG
HighDeltaPOST   737  CTCAGCCTCCGGAGTAGCTAGGACTACAGGCGCGTGTCACCATGTCCCAG
LowDeltaPOST    745  CTCAGCCTCCGGAGTAGCTAGGACTACAGGCGCGTGTCACCATGTCCCAG

                               810       820       830       840       850
                     ....|....|....|....|....|....|....|....|....|....|
Reference       795  CTAATCTTTCTTTATTTTTAGTAGAGACGTGTTAGCCAGGTTTCACCGTG
HighDeltaPOST   787  CTAATCTTTCTTTATTTTTAGTAGAGACGTGTTAGCCAGGTTTCACCGTG
LowDeltaPOST    795  CTAATCTTTCTTTATTTTTAGTAGAGACGTGTTAGCCAGGTTTCACCGTG

                               860       870       880       890       900
                     ....|....|....|....|....|....|....|....|....|....|
Reference       845  TTACCGTGTTACCGTATTCCAGCCGGGCTCCGCCCCTGGATTCGGTGGAC
HighDeltaPOST   837  TTACCGTGTTACCGTATTCCAGCCGGGCTCCGCCCCTGGATTCGGTGGAC
LowDeltaPOST    845  TTACCGTGTTACCGTATTCCAGCCGGGCTCCGCCCCTGGATTCGGTGGAC

                               910       920       930       940       950
                     ....|....|....|....|....|....|....|....|....|....|
Reference       895  AGACCAGCTTTACCTCCAGAGACTCCCCCTGTCTTCCCTGCACCGGCTGA
HighDeltaPOST   887  AGACGAGCTTTACCTCCAGAGACTCCCCCTGTCTTCCCTGCACCGGCTGA
LowDeltaPOST    895  AGACCAGCTTTACCTCCAGAGACTCCCCCTGTCTTCCCTGCACCGGCTGA
```

```
                               960       970       980       990      1000
                           ....|....|....|....|....|....|....|....|....|....|
Reference         945      CTATACCTACACACTTCGGTGGCACAATCTTTGTTTAGATATGCTGATGC
HighDeltaPOST     937      CTATACCTACACACTTCGGTGGCACAATCTTTGTTTAGATATGCTGATGC
LowDeltaPOST      945      CTATACCTACACACTTCGGTGGCACAATCTTTGTTTAGATATGCTGATGC

                              1010      1020      1030      1040      1050
                           ....|....|....|....|....|....|....|....|....|....|
Reference         995      CTTCCTTCGCTGGGAACGGTCCAGTCCTGCCCGCCTGGCGCTCAGCTCTG
HighDeltaPOST     987      CTTCCTTCGCTGGGAACGGTCCAGTCCTGCCCGCCTGGCGCTCAGCTCTG
LowDeltaPOST      995      CTTCCTTCGCTGGGAACGGTCCAGTCCTGCCCGCCTGGCGCTCAGCTCTG

                              1060      1070      1080      1090      1100
                           ....|....|....|....|....|....|....|....|....|....|
Reference         1045     CGGATCTCAGGGGATGGAGTGGCAATCTCATTGCTCCCGCCTCTCCTGAC
HighDeltaPOST     1037     CGGATCTCAGGGGATGGAGTGGCAATCTCATTGCTCCCGCCTCTCCTGAC
LowDeltaPOST      1045     CGGATCTCAGGGGATGGAGTGGCAATCTCATTGCTCCCGCCTCTCCTGAC

                              1110      1120      1130      1140      1150
                           ....|....|....|....|....|....|....|....|....|....|
Reference         1095     CCCTCTCGCCTTCTCCTCGAGACCCGGGTTAAGTGGGCAGAGGGCTCCGA
HighDeltaPOST     1087     CCCTCTCGCCTTCTCCTCGAGACCCGGGTTAAGTGGGCAGAGGGCTCCGA
LowDeltaPOST      1095     CCCTCTCGCCTTCTCCTCGAGACCCGGGTTAAGTGGGCAGAGGGCTCCGA

                              1160      1170      1180      1190      1200
                           ....|....|....|....|....|....|....|....|....|....|
Reference         1145     GGGTCCTCTCCCGCCGTGGGATTGCACAGAGGGCTGGGTCCGCAGGCTGG
HighDeltaPOST     1137     GGGTCCTCTCCCGCCGTGGGATTGCACAGAGGGCTGGGTCCGCAGGCTGG
LowDeltaPOST      1145     GGGTCCTCTCCCGCCGTGGGATTGCACAGAGGGCTGGGTCCGCAGGCTGG

                              1210      1220      1230      1240      1250
                           ....|....|....|....|....|....|....|....|....|....|
Reference         1195     CTAAAAGGACCTAGCCCAGGATTGCGAGGCAGCGCCCTCCCTGCAGCTGG
HighDeltaPOST     1187     CTAAAAGGACCTAGCCCAGGATTGCGAGGCAGCGCCCTCCCTGCAGCTGG
LowDeltaPOST      1195     CTAAAAGGACCTAGCCCAGGATTGCGAGGCAGCGCCCTCCCTGCAGCTGG

                              1260      1270      1280      1290      1300
                           ....|....|....|....|....|....|....|....|....|....|
Reference         1245     ACAGACTCGGGCGCCCGGCCCAAAGCCCGGCAGGGGCGTGTCCGCGCGCT
HighDeltaPOST     1237     ACAGACTCGGGCGCCCGGCCCAAAGCCCGGCGGGGGCGTGTCCGCGCGCT
LowDeltaPOST      1245     ACAGACTCGGGCGCCCGGCCCAAAGCCCGGCAGGGGCGTGTCCGCGCGCT

                              1310      1320      1330      1340      1350
                           ....|....|....|....|....|....|....|....|....|....|
Reference         1295     CACCTGCTATTGGCCAGGTGGGGCTGTCGGCTGCCAGCAGTGCTTATGCT
HighDeltaPOST     1287     CACCTGCTATTGGCCAGGTGGGGCTGTCGGCTGCCAGCAGTGCTTATGTT
LowDeltaPOST      1295     CACCTGCTATTGGCCAGGTGGGGCTGTCGGCTGCCAGCAGTGCTTATGCT

                              1360      1370      1380      1390
                           ....|....|....|....|....|....|....|....|..
Reference         1345     TTAAGTGCGGAGCGGGTGGCTGCGGAGCCAGGCGCGGCGCAG
HighDeltaPOST     1337     TTAAGTGCGGAGCGGGTGGCTGCGGAGCCAGGCGCGGCGCAG
LowDeltaPOST      1345     TTAAGTGCGGAGCGGGTGGCTGCGGAGCCAGGCGCGGCGCAG
```
