# Supplementary material for: Genome-Wide Meta-Analysis of Homocysteine and Methionine Metabolism Identifies Five One Carbon Metabolism Loci and a Novel Association of ALDH1L1 with Ischemic Stroke
Source: PLoS Genet. 2014 Mar 20;10(3):e1004214. doi: 10.1371/journal.pgen.1004214 (PMC3961178; doi:10.1371/journal.pgen.1004214)
Supplement: Table S1 — Independent GWAS results for ΔPOST phenotype in VISP and FHS. (PDF) [file pgen.1004214.s006.pdf]

Study	RS#	Chromosome	Position (bp)	Associated Gene	Gene Region	Alleles (Minor/Major)	MAF	N	SE	P-value
**VISP**										
	rs1129186	6	42932202	*GNMT/PEX6*	*GNMT* 3' / *PEX6* Exon 1	C/T	0.546	2100	0.031	$2.47x10^{-23}$
	rs234714	21	44488033	*CBS*	Intron 4 /5' UTR	T/C	0.223	2100	0.037	$1.04x10^{-9}$
**FHS**										
	rs9471968	6	42905235	*CNPY3/GNMT*	*CNPY3* Intron 3 / *GNMT* 5'	A/G	0.466	2710	0.026	$7.35x10^{-43}$
	rs234714	21	44488033	*CBS*	Intron 4 / 5' UTR	T/C	0.201	2710	0.046	$2.29x10^{-18}$
	rs1047891	2	211540507	*CPS1*	Exon 37 missense Ser (ACC) / Phe (AAC)	A/C	0.306	2710	0.034	$1.35x10^{-8}$
	rs2364368	3	125905080	*ALDH1L1*	5'	T/A	0.425	2710	0.027	$4.04x10^{-13}$
	rs4948102	7	56097265	*PSPH*	Intron 3	C/G	0.249	2710	0.035	$1.40x10^{-15}$
