# Supplementary material for: Genome-Wide Meta-Analysis of Homocysteine and Methionine Metabolism Identifies Five One Carbon Metabolism Loci and a Novel Association of ALDH1L1 with Ischemic Stroke
Source: PLoS Genet. 2014 Mar 20;10(3):e1004214. doi: 10.1371/journal.pgen.1004214 (PMC3961178; doi:10.1371/journal.pgen.1004214)
Supplement: Table S2 — Correlation coefficients of principal components and ΔPOST. As an exploratory analysis, we calculated the Pearson correlation coefficients of ΔPost with all the covariates (age, first 10 principal components from EIGENSTRAT analysis (FHS) or KING (VISP)) and corresponding p-values in FHS and VISP. (PDF) [file pgen.1004214.s007.pdf]

FHS

Covariate	AGE	PC1	PC2	PC3	PC4	PC5	PC6	PC7	PC8	PC9	PC10
**∆POST Correlation**	-0.055	-0.047	-0.016	0.003	0.027	0.009	0.034	-0.016	0.008	-0.004	0.012
**P-value**	0.004	0.015	0.42	0.87	0.16	0.63	0.078	0.41	0.69	0.85	0.52

VISP

Covariate	AGE	PC1	PC2	PC3	PC4	PC5	PC6	PC7	PC8	PC9	PC10
**∆POST Correlation**	-0.045	-0.111	-0.010	0.057	-0.008	-0.004	-0.017	0.011	0.039	-0.020	0.004
**P-value**	0.0467	<.0001	0.6586	0.0127	0.727	0.8746	0.4577	0.6301	0.0911	0.3735	0.8533
